# Atypical suicide attempt facilitated by levodopa in a patient with impending Parkinson’s Disease masquerading as a mood disorder: a case report

**DOI:** 10.1192/j.eurpsy.2024.1636

**Published:** 2024-08-27

**Authors:** A. Mara, S. Myriknas, S. Tychala

**Affiliations:** Psychiatric Hospital, Thessaloniki, Greece

## Abstract

**Introduction:**

Parkinson’s Disease (PD) is a neuropsychiatric disorder whose diagnosis is mainly based on motor impairment. However, increasing evidence suggests that neurodegeneration precedes the appearance of motor disturbances to manifest itself with hyposmia, sleep, and affective disorders. The disease’s insidious onset and comorbidity with psychiatric symptoms require specialized knowledge and delicate pharmacological maneuvers to provide the patient with the best possible treatment at the most precise moment. Studies have also highlighted the potential increase in impulsivity patients may experience upon initiation with levodopa.

**Objectives:**

Τo raise awareness of the complexity of treating patients with PD that also face psychiatric comorbidities that appeared before the motor symptoms, including preoccupation with death, and highlight the need for intensive interdisciplinary medical follow-up of such patients.

**Methods:**

We report a clinical case of a 54-year-old man who was admitted to the psychiatric emergency department after a suicide attempt by self-inflicting severe bilateral neck, wrists, and femoral triangles injuries, as well as self-cutting his Achilles tendon. The patient had a history of a one-year mixed anxiety and depressive disorder and was treated on an outpatient basis with amitriptyline/perphenazine (10+2)mg, sulpiride 50mg, and clonazepam 2mg. One month before his attempt, the patient started experiencing unilateral upper and lower limb rigidity with bradykinesia and “pill-rolling” resting tremor of the same hand and was prescribed levodopa/benserazide (200+50)mg three times per day. After two days of starting the new medication, the patient attempted suicide by the method mentioned above.

**Results:**

After surgical assessment and care, the patient recovered at the psychiatric department for 21 days and was treated with sertraline 50mg, which was later increased to 100mg. As an adjunctive treatment, the patient also received mirtazapine 15mg/day, quetiapine 200mg/day, and lorazepam 3mg/day. On the 15th day of his hospitalization and after a neurological assessment, the patient was started on levodopa/benserazide (200+50)mg one-quarter three times per day. At discharge, he presented significant clinical improvement regarding both his mental health and neurologic somatic symptoms.

**Conclusions:**

Patients with PD require a multidisciplinary approach by a trained medical team. Clinicians should titrate dopamine replacement agents with caution, especially for those experiencing mood disorders, because they might increase the patient’s impulsivity, “assisting” a depressive patient with suicidal ideation to finally commit suicide.

**Disclosure of Interest:**

None Declared

